# Creation of knowledge‐based planning models intended for large scale distribution: Minimizing the effect of outlier plans

**DOI:** 10.1002/acm2.12322

**Published:** 2018-04-06

**Authors:** Jorge Edmundo Alpuche Aviles, Maria Isabel Cordero Marcos, David Sasaki, Keith Sutherland, Bill Kane, Esa Kuusela

**Affiliations:** ^1^ CancerCare Manitoba 675 McDermot Ave. Winnipeg MB R3E 0V9 Canada; ^2^ University of Manitoba Winnipeg MB R3T 2N2 Canada; ^3^ Varian Medical Systems Paciuksenkatu 21 00270 Helsinki Finland

**Keywords:** DVH estimation, Knowledge‐based planning, outliers, radiation therapy, machine learning.

## Abstract

Knowledge‐based planning (KBP) can be used to estimate dose–volume histograms (DVHs) of organs at risk (OAR) using models. The task of model creation, however, can result in estimates with differing accuracy; particularly when outlier plans are not properly addressed. This work used RapidPlan^™^ to create models for the prostate and head and neck intended for large‐scale distribution. Potential outlier plans were identified by means of regression analysis scatter plots, Cook's distance, coefficient of determination, and the chi‐squared test. Outlier plans were identified as falling into three categories: geometric, dosimetric, and over‐fitting outliers. The models were validated by comparing DVHs estimated by the model with those from a separate and independent set of clinical plans. The estimated DVHs were also used as optimization objectives during inverse planning. The analysis tools lead us to identify as many as 7 geometric, 8 dosimetric, and 20 over‐fitting outliers in the raw models. Geometric and over‐fitting outliers were removed while the dosimetric outliers were replaced after re‐planning. Model validation was done by comparing the DVHs at 50%, 85%, and 99% of the maximum dose for each OAR (denoted as V50, V85, and V99) and agreed within −2% to 4% for the three metrics for the final prostate model. In terms of the head and neck model, the estimated DVHs agreed from −2.0% to 5.1% at V50, 0.1% to 7.1% at V85, and 0.1% to 7.6% at V99. The process used to create these models improved the accuracy for the pharyngeal constrictor DVH estimation where one plan was originally over‐estimated by more than twice. In conclusion, our results demonstrate that KBP models should be carefully created since their accuracy could be negatively affected by outlier plans. Outlier plans can be addressed by removing them from the model and re‐planning.

## 1 INTRODUCTION

Knowledge‐based planning (KBP) is an emerging field in radiation therapy which uses machine learning techniques to estimate radiation therapy dose. KBP can be generalized to be the automation of different steps in the creation of a plan based on past practice. These steps can range from the estimation of field direction,^1^ weights of optimization objectives,^2^ and even dose distribution.^3^, ^4^ The majority of KBP work, however, has focused on estimating dose–volume histograms (DVHs)^5^, ^6^, ^7^, ^8^, ^9^ which are commonly used to evaluate plan quality and guide the inverse planning process.

Radiation treatment planning is a complex process which can result in an infinite number of plans; some of which are suboptimal. This is because the final dose distribution is dependent on the geometry of the organs at risk (OAR) with respect to the target. Other factors that can potentially affect the quality of the final plan are differences in dose prescription,^10^ treatment technique, and planner experience.^11^ It is because of these reasons that plan quality evaluation has been based on user experience primarily making the development of quantitative tools necessary.

KBP tools have been in development by different groups over the past few years. Wu et al. introduced the concept of the overlap volume histogram and used it to estimate the DVHs for OARs,^6^ and automate the treatment planning process in head and neck (HN) cases.^12^ Zhu et al. used the distance to target histogram, support vector regression, and principal component analysis to estimate DVHs in the context of adaptive radiation therapy.^7^ Yuan et al. proved that it is possible to quantify the complex relationship that different factors have on the final shape of the DVH.^9^ This group also used their tool to exchange models that summarize plan creation strategies among different institutions, hence providing a means to standardize treatment planning.^13^, ^14^ Moore et al. introduced a KBP tool to perform quality assurance on intensity modulated radiation therapy (IMRT) plans and reduce dosimetric variability.^15^ Appenzoler et al. described a mathematical framework to estimate differential DVHs using a summation of skew‐normal distributions whose parameters were fitted based on previous plans.^8^ This work has being further expanded to be used in the case of intracranial lesions.^5^

The work described in the previous paragraph has been done using tools developed in house. It is only more recently that a commercial application became available (RapidPlan^™^, Varian Medical Systems, Palo Alto, CA). RapidPlan allows the user to estimate DVHs of OARs using “models” which are trained using principal component analysis (PCA) and stepwise regression analysis. This training requires the user to define a number of plans as the training set which can be done in a number of ways, hence necessitating model performance evaluation. Fogliata et al. have evaluated the performance of RapidPlan using volumetric arc therapy in hepatocellular, lung, and prostate cancer.^16^, ^17^ Their results showed that RapidPlan models can be used to achieve clinically acceptable plans. Tol et al. evaluated the performance RapidPlan on head and neck and showed the ability to achieve clinically acceptable plans for this site.^18^ These studies identified the need to investigate the proper identification of outlier plans, that is, plans that do not seem to follow the general trend of the training set and could have a negative effect on the models. Delaney et al. systematically introduced dosimetric outliers in KBP models and found little change in the accuracy of the model.^19^ This was attributed to the decreased precision of the estimated DVHs, whose lower boundary was used for planning. Their study focused on the effect of dosimetric outliers and briefly mentions the negative effect of geometric outliers on KBP models. Proper outlier analysis also helps control model over learning, meaning that the trained model only reflects the training set and not similar cases.

This manuscript describes the process used to create models to be used by a wide range of users with emphasis on the steps taken to address all types of outliers. This is the first study, to the best of our knowledge, which categorizes the different types of outliers, provides strategies to address each of them as well as examples of their effect in the model. This extended outlier analysis will benefit users who are starting to build their own KBP models. Furthermore, the models created are included in the commercial distribution of RapidPlan and we believe it is important for the user to understand the philosophy used to create the models as well as the accuracy obtained during their creation. We will show that proper outlier removal can affect the accuracy of estimated DVHs. This outlier analysis becomes particularly important when creating models that have the potential to be used by a wide range of users.

## 2 MATERIALS AND METHODS

### 2.A Overview of the algorithm

The commercial implementation of KBP uses a DVH estimation algorithm that is different from the algorithms described above. This algorithm estimates DVHs by dividing the OAR volume into four different regions: the out‐of‐field, in‐field, leaf‐transmission, and overlap region. All regions contribute to the final DVH but each region is modeled differently depending on the desired detail and accuracy. While both the in‐field and overlap regions are dependent on fluence modulation, the shapes of DVHs in the overlap region are similar across all plans. For this reason, and under the assumption that the target is the primary priority in that area, the overlap region is modeled by the mean and standard deviation of the DVHs part corresponding to that overlap area. In the in‐field region, however, the shapes of the in‐field DVHs vary considerably across all the plans. It is in this region where the major improvements in tissue sparing could be achieved, under the assumption that the target is the primary priority in the overlap area. Thus, this in‐field region, where higher modulation is present and higher accuracy is desired, is modeled using principal component analysis (PCA) and regression techniques. PCA is used to transform the histograms into principal component scores (PCS), thus reducing the dimensionality of the problem. PCA is also used to parametrize the geometry‐based expected (GED) histogram, a 3D matrix incorporating the target geometry, target dose, and treatment field geometry. Other parameters that affect the PCS of the DVH include the OAR volume, target volume, OAR overlap volume percentage to target, and the proportion of the OAR that is out‐of‐field.*

### 2.B Models creation

#### 2.B.1 Training plans selection

Two models were created, one for prostate, and one for head and neck. Once the anatomical sites were identified, patients who received treatments to these sites were retrospectively selected by searching our institution database. Using previously treated plans was desirable since these plans reflect treatment techniques that are clinically acceptable at our institution.

The models were trained with plans selected to include a wide range of cases found commonly at our clinic. More specifically, prostate treatments included:

a Low‐ and intermediate‐risk patients. These patients include those whose clinical target volume (CTV) consists of the prostate with or without the seminal vesicles. These patients are treated with a prescription dose of 78 Gy.
b Post‐op patients. Patients whose CTV is defined as the Prostate Bed. The prescription dose for this group of patients was 66 Gy.
c High‐risk patients. Patients whose prostate or prostate bed and pelvic lymph nodes were treated. These groups of patients are treated using a sequential boost technique. The CTV of the first phase includes the prostate (or prostate bed) and the pelvic lymph nodes. The prescription dose for this portion of treatment is 46 Gy. The CTV of the second phases is that of the two categories described above but with prescription doses of 32 Gy or 20 Gy.

The training plans used to create the HN models consisted of all types of HN plans treated at our center. The selection of HN plans was limited to those treated using volumetric arc therapy (RapidArc^®^, Varian Medical Systems, Palo Alto, CA). This was done in order to minimize the number of variables that could affect the behavior of the models; for example, differences in treatment techniques (static field IMRT vs RapidArc). This resulted in a total of 177 plans since the implementation of this technique at our center. Among these 177 plans, 61 plans were assigned to the validation set (see below) and the reminder 116 plans to the training set. These training plans included a wide range of HN clinical cases; such as nasopharynx, oropharynx, and post‐op treatments. Table 1 summarizes the number of HN plans used to create the model in terms of number of targets and dose prescription combinations.

**Table 1:** Details of the plans used to train the HN model

Number of targets	Number of plans used for training	Dose (Gy) prescription's combinations
3	62	(70,63,56), (70,59,54), (66,60,54), (66,59,56)
2	35	(70,63), (66,60), (66,56), (60,54), (50,45)
1	19	60, 50

All Prostate plans were treated using RapidArc^®^ with two full arcs. HN treatments were treated using two partial or full arcs depending on whether the disease was unilateral or bilateral respectively.

#### 2.B.2 Model creation process

The prostate model was created by including prostate plans from all categories (listed in Section 2.B.1) and following a step‐by‐step process. This process consisted of creating a general prostate model and category‐specific models in parallel. The first model consisted of 20 low/intermediate‐risk patients plans (all with a prescription dose of 78 Gy). Twenty plans were used since this was the minimum number of plans that were allowed in order to create the model. A second model, specific to the second phase high‐risk patients plans, was created using 20 additional plans (19 of which had a prescription dose of 32 Gy and one with a prescription dose of 20 Gy). These plans were added to the previously created model resulting in a general model with 20 Intact prostate plans and 20 second phase plans. Fifteen post‐op specific were then added to the general model, all with prescription doses of 66 Gy. This resulted in a general model consisting of 20 intact prostate plans, 15 post‐op plans, and 20 second phase plans. Lastly, 43 plans whose CTV included the pelvic lymph nodes were added to the general model.

The HN model was created following a different procedure from the one followed to create the prostate model. Instead of adding plans to the model in increments, an initial general model was created with the entire training set of HN plans available (*N* = 116). This approach was followed since it was unclear how HN plans should be categorized. Even for treatments of the same site (e.g., oropharynx); there were multiple combinations of dose prescription, changes in the relative geometry of the target with respect to OARs, and number of targets. Using the largest number of plans available, and using scatter plots to identify clusters resulted in a more efficient (and practical) approach.

The models were evaluated using the coefficient of determination and the chi‐squared test. In order to recognize potential outliers, the scatter plots (provided by the RapidPlan^™^ algorithm) were used to detect cases not fitting to the general model behavior. Scatter plots show the relationship between the dependent variable DVH PCS on independent variables and any training case appearing to be exceptionally far away from the regression line was considered as a potential outlier. In addition, potentially influential cases were determined using Cook's distance (the threshold value being roughly 3.0). Cook's distance was used since it provides a measure of influence of an outlier by omitting the plan from the regression analysis.^20^ Being influential means that the single case has a large impact for the regression line and does not necessarily appear as an outlier in the scatter plot. Other metrics provided by RapidPlan^™^, such as modified Z scores, studentized residual, etc., were mainly omitted since the same information was available in the scatter plots. The influence of the potential outliers was tested by removing them from the training set and re‐training the model. The removal of structures was done iteratively by excluding one or two strongest influential cases (or outliers) at a time and monitoring the improvement in the trained model. The removal stopped once no more significant improvement was observed. The chi‐squared test was used to monitor over‐fitting. A threshold value of 1.3 was used as an indication of no severe over‐fitting.

#### 2.B.3 Outlier analysis

Outliers are plans which deviate from the general trend in the analysis. Their effect on a model is typically not immediately clear because not all outliers affect the overall trend of the data in the regression analysis.

The first step in addressing identified outlying plans consisted of re‐planning them. An alternative approach would consist of removing these plans from the model entirely. However, this does not ensure that the model will work for these patients in the future. Re‐planning also helps to reduce the uncertainty (width of the estimate band) in the estimate. Care was taken to ensure that dose reduction to an OAR of interest did not result in an increased dose to another OAR nor reduce target coverage. Treatment plans for which re‐planning was able to reduce the dose effectively are *dosimetric outliers* and the outlying plans were replaced by the new plans. Treatment plans for which re‐planning was unable to reduce the dose for a given OAR are *geometric outliers*. Geometric outliers are plans were one or more OARs differ geometrically from the rest of the training set and were addressed in two different ways. Geometric outliers with large Cook's distances were removed from the model since they can negatively affect the model for the majority of plans. Geometric outliers which are non‐influential were kept in the model as they do not affect the model in a negative way and may provide useful information for the model to estimate DVHs in plans with similar properties.

*Over‐fitting* means that the estimation model only describes the training set but may not generalize well for other cases. Outliers that cause over‐fitting are a special type of outliers and occurs when a single plan increases the number of variables needed to predict the DVHs of the training set. Over‐fitting was evaluated by inspecting all of the scatter plots for each OAR in conjunction with the goodness of fit and chi‐squared metrics. The ideal way to address this type of outliers is to find plans with similar characteristics and add them to the training set. Finding similar plans is challenging (or potentially impossible) and, hence, plans that caused over‐fitting were removed from the model.

### 2.C Models validation

The validation process consisted of using the trained models to estimate DVHs on a group patients with similar characteristics of those used to train the model. The purpose of the validation process is to provide confidence that the model does not estimate the DVH variation in the training set only. The validation set of patients were completely independent from the patients used for training. These patients had undergone treatment at our clinic, and so had clinically acceptable plans. The DVHs of the clinical plans in the validation patients were compared against the estimated DVHs obtained from the model. Estimated DVHs were subtracted from the clinical DVHs at points corresponding to 50%, 85%, and 99% of OAR maximum dose (which we will refer to a V50, V85, and V99). Yuan et al.^9^ quantified this difference with respect to the prescription dose but these metrics would become irrelevant for OARs whose dose is less than 50% of the prescription dose. Instead, the doses used to compare DVHs were normalized with respect to the maximum OAR dose. Note that the V50, V85, and V99 metrics used in this study approach those of Yuan et al. in cases of the bladder and rectum where the OAR maximum dose approaches the prescription dose allowing for direct comparison. The mean differences at V50, V85, and V99 were calculated by averaging over all validation patients along with the standard error of the mean.

The validation patients used to validate the prostate model consisted of 20 low/intermediate‐risk patients (ten plans whose CTV included the seminal vesicles and ten plans without seminal vesicles), ten post‐op cases, and ten cases corresponding to the second phase of high risk patients.

As mentioned above, the HN model was created by retrospectively identifying a large number of HN plans. The group of patients used to validate the HN model was chosen according to the following criteria. HN plans whose dose prescription was used once, was assigned as a validation patient since it is desirable to test the model in most clinical scenarios. Plans with the same dose prescriptions were split into training and validation according to the laterality (left, right, and bilateral), region in the body (superior, middle, and inferior part of the HN region), and number of targets. This resulted in a total of 61 HN validations patients for the HN model with the number of targets and dose prescriptions as indicated in Table 2 below.

**Table 2:** Summary of plans used to validate the HN model including different combinations of dose prescriptions

Number of Targets	Number of plans	Dose (Gy) prescription's combinations
3	26	(70,63,56), (70,59,54), (70, 59, 50), (70,56,52), (66,59,56), (66,60,54)
2	16	(70,63), (70,60), (66,60), (66,56), (66,54), (60,54), (50,45)
1	19	70, 66, 60, 50, 36, 24

Both models were used to create plans as part of the validation process. The lower boundary of the estimated DVHs were set as optimization objectives with fixed priorities for all validation plans. These are called line objectives and were, arbitrarily, set with a priority value of 50 for the bladder, rectum, and femoral heads. Two point objectives were set for the planning target volume (PTV) with a priority of 120: a lower V98% = 100% and an upper V102% = 0%. The full set of optimization objectives used in the HN model is too large to be displayed in this manuscript given the large variability of HN treatments For example, different clinical metrics are requested depending on the location of the tumor and, hence, the initial optimization objectives of untrained structures. The optimization objectives of trained OARs were always the same and the full set of optimization objectives can be found in the model data sheet.† In summary, line optimization objectives with a priority of 40 were used for the brain, brainstem, cord, cord PRV, mandible, and oral cavity. A priority value of 65 was given to the line objective of the parotids following the importance that is given to this OAR at our institution. Three point objectives were used for each target of HN plans: (a) a lower V100% = 100% with priority of 130, (b) an upper V101% <= 15% with priority of 100, and (c) an upper V102% = 0 with priority of 130. A normal tissue objective was also used during optimization with a priority equal to 100 for the prostate model and 80 for the HN model.

The optimization was run without any user interaction and the final plans were compared against the original clinical plans. These runs were done automatically by two of the authors who are independent to the institution where the plans were created.

## 3 RESULTS

### 3.A Model training results

#### 3.A.1 Prostate model

**Figure 1:**
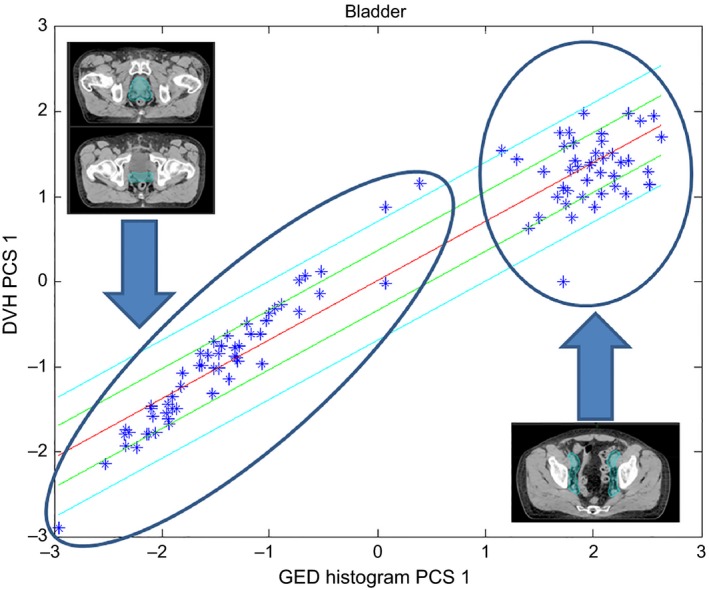
Scatter plot for the bladder including all types of prostate plans. Points encircled in the left ellipse represent plans whose CTV excludes the pelvic lymph nodes while those on the right ellipse include the lymph nodes (as shown by the contours on the inserted figures).

Figure 1 shows a scatter plot for the bladder obtained with all categories of prostate plans. The figure shows the dependence of the DVH 1st principal component score and the GED histogram 1st principal component score. The units of the graphs are dimensionless. Two ellipses are also shown to encircle the clusters that make this data bipolar. The plans enclosed by the ellipse on the right of the graph correspond to those whose CTV include the pelvic lymph nodes while all the reminder of the categories are encircled on the ellipse on the left. Forty‐three plans were added since a higher number of plans were necessary in order to obtain an evident cluster. Figure 1 also shows the line fitted by the model along with the lines corresponding to one and two standard deviations. It can be seen that the line fitted by the model results in a compromise. Given that treatments involving pelvic lymph nodes are substantially different from the other types of prostate plans, these types of plans were excluded from the general prostate model and a model‐specific for this category would have to be created. Once pelvic lymph nodes plans were removed, the bladder scatter plot showed that the reminder categories of prostate plans followed the same trend.

**Figure 2:**
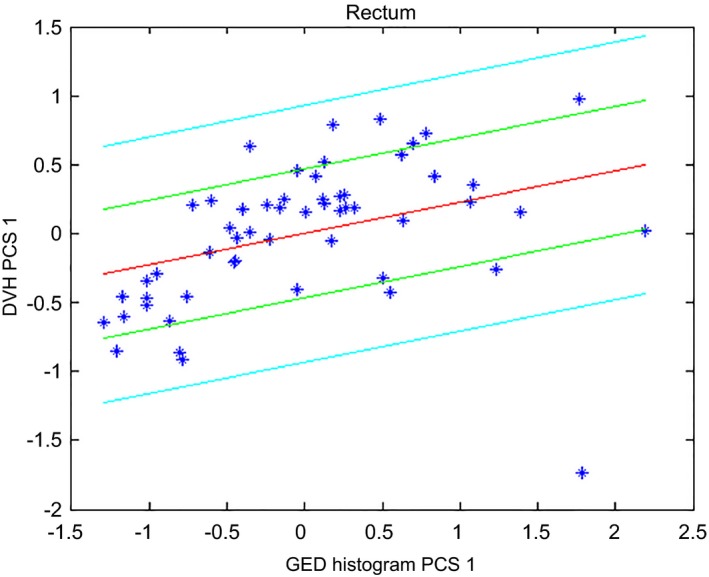
Scatter plot for the rectum of the prostate model after excluding high‐risk patients from the model.

Table 3 lists the values of the metrics used to evaluate the goodness of statistics for the first and final models. Figure 2 shows the scatter plot for the rectum where a wider spread can be observed (also reflected on the regression model's coefficient of determination) compared to that of the bladder. Twenty more plans (8 low/intermediate‐risk cases, 6 second phase cases, and 6 Post‐op cases) were added to improve the statistics for the regression of this structure. The decreased values for both coefficient of determination and chi‐squared test for the final model reflect the reduction in over‐fitting (chi‐squared test results closer to 1 correspond to better fits^16^).

**Table 3:** Statistical metrics for the first and final prostate models

Structure	Regression model coefficient of determination		Regression model's parameter average chi‐squared	
	First model	Final model	First model	Final model
Bladder	0.934	0.914	1.068	1.031
Rectum	0.756	0.671	1.150	1.086
Femoral heads	0.942	0.919	1.068	1.057

**Figure 3:**
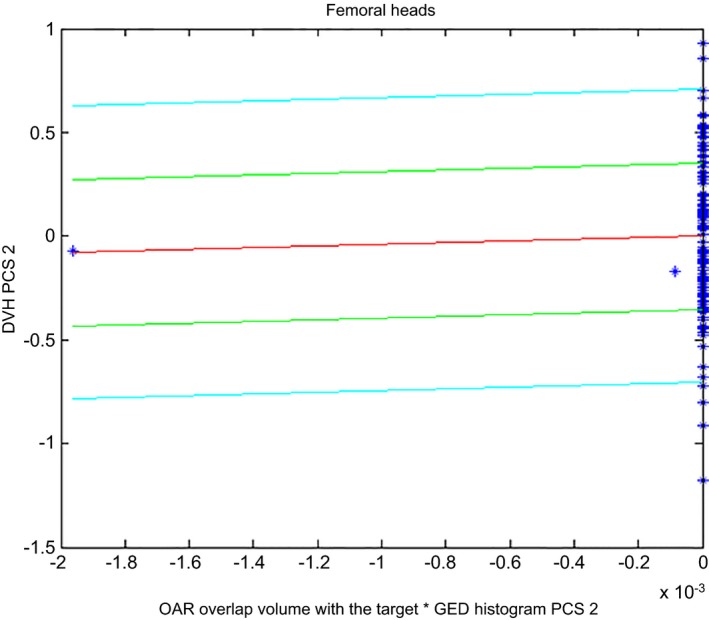
Scatter plot for the femoral heads showing the presence of over‐fitting.

**Figure 4:**
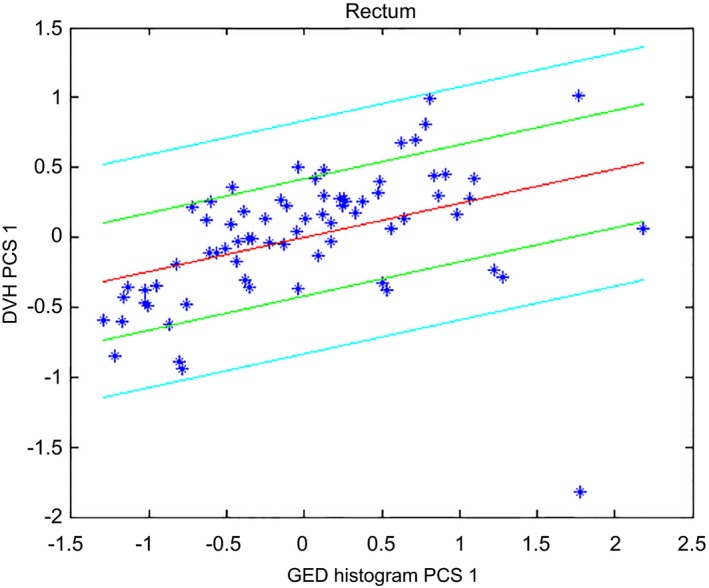
Scatter plot for the rectum. The figure shows a geometric outlier in the lower right corner which was eventually removed.

Three plans were completely removed from the model and the following paragraph explains the reasons. Figure 3 below shows the scatter plot for the femoral heads and the first plan that was removed (shown as the data point on the far left) because it was causing over‐fitting. This plan also flagged as an outlier in terms of the “OAR overlap volume percentage to target” and does not result in an increase on the chi‐square for this structure by itself. The second plan that was completely removed is shown in the bottom right corner of Fig. 4. The plan increases the maximum value of Cook's distance to 500 for the rectum compared to 27.7 in its absence. This plan consisted of a geometric outlier and was therefore removed from the model. The third plan that was removed flagged as a geometric outlier from the point of view of the “OAR overlap volume percentage to the target”.

**Figure 5:**
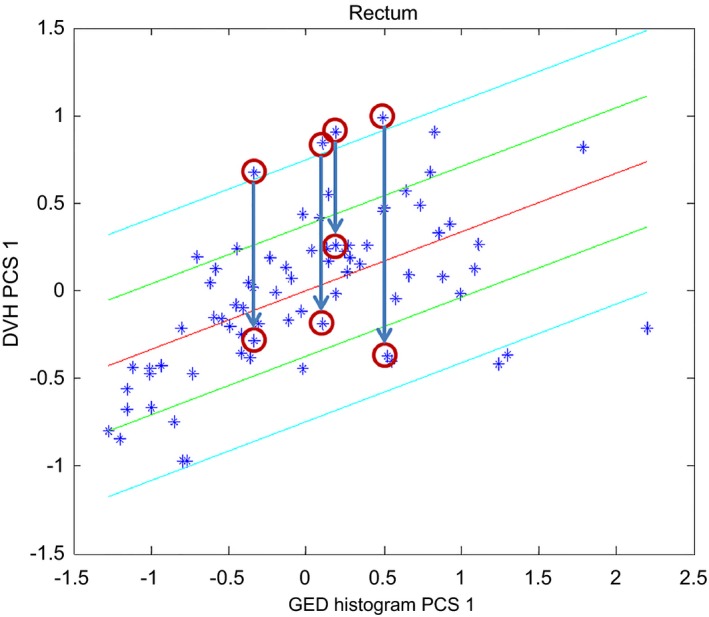
Scatter plot for the rectum showing the effect of re‐planning dosimetric outliers. The original plans (highlighted by four circles at the beginning of the arrows) were replaced by the new plans (highlighted by the circles under the arrows).

Four dosimetric outliers plans were re‐planned in order to reduce the dose to the rectum without compromising the other structures of interest. Figure 5 below shows the rectum scatter plots including the original clinical plans and the plans obtained after re‐planning. This figure shows that these plans were receiving doses larger than those received by the majority of the plans. The original clinical plans were replaced by the re‐plans.

The final version of the prostate model consisted of a total of 72 training patients: 27 intact prostate plans (10 without seminal vesicles and 17 with seminal vesicles), 19 post‐op plans and 26 second phase plans (25 intact prostate cases and 1 post‐op case).

#### 3.A.2 HN model

**Figure 6:**
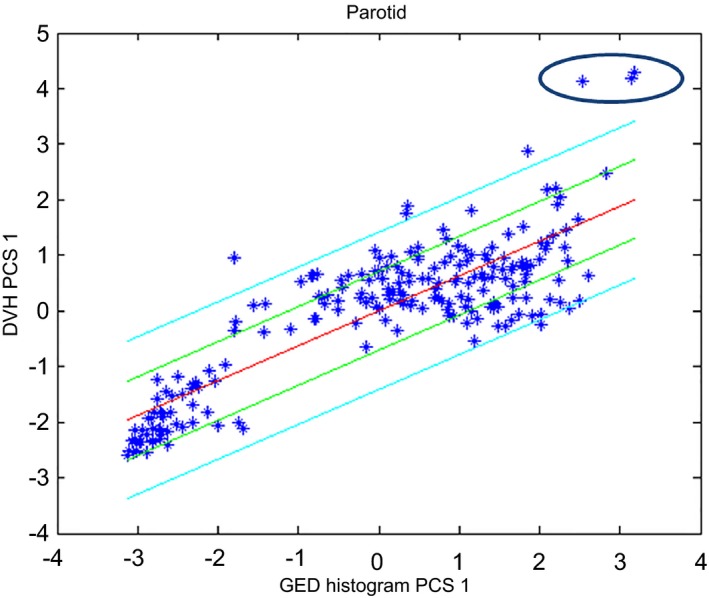
Scatter plot for the parotids for the first HN model. Plans whose parotids overlapped with the target are encircled in the upper right corner.

**Figure 7:**
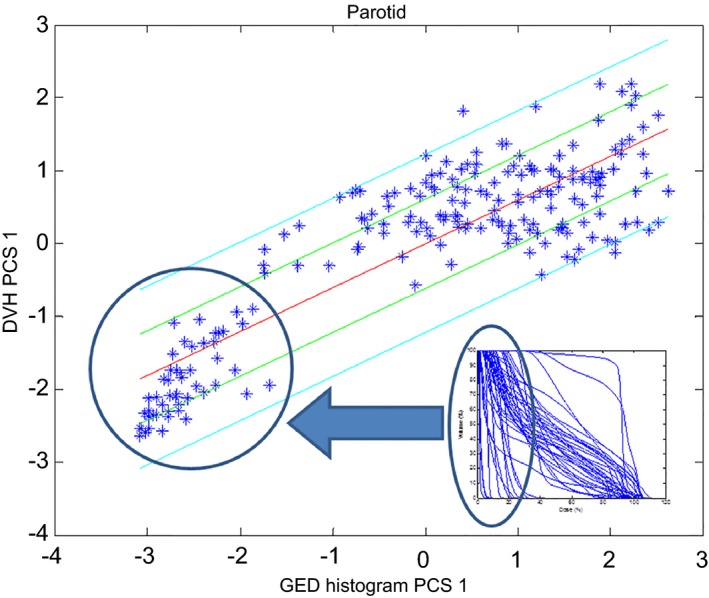
Parotids scatter plot for the final HN model. The figure shows an insert with parotids DVHs to illustrate the location of contralateral parotids on the scatter plot.

Figure 6 shows the scatter plot that was initially obtained for the parotids (laterality combined as a single training structure, similarly to the femoral heads in the prostate model). Figure 7 shows the corresponding scatter plot that was obtained after two plans were re‐planned (dosimetric outliers) and the parotids for three plans were excluded (influential geometric outliers). Further investigations showed that these three outliers, encircled in the upper right corner, corresponded to plans with large overlaps of the parotids with the targets. In fact, similar plans in the validation set showed that these plans resulted in a third cluster (tri‐polar data). These three plans were then removed since bi‐polar and tri‐polar data are better described by separate models. The third group of patients was excluded from the model and, instead, the model was validated including these types of plans to investigate its performance under these circumstances. Figure 7 shows the presence of two clusters corresponding to contralateral parotids (grouped in the lower left corner) and the remainder of the parotids. While the accuracy of the model can be improved by creating separate models for these two groups, this would require the user to decide which model to use. This approach is not possible if the proper model cannot be determined upfront, as was the case here, and so it was decided to merge both parotids into the same model and evaluate the accuracy resulting from this compromise. Note that this approach is different from that followed to create the prostate model, where a separate model would have to be created for high risk patients which can be easily identified.

**Figure 8:**
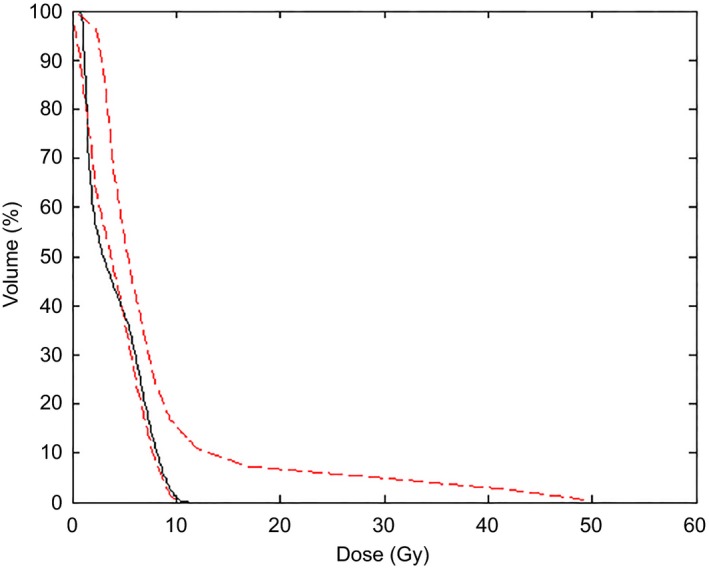
Clinical (black solid line) and estimated (red‐dashed lines) DVHs for a contralateral parotid. The figure shows the effect of merging the two clusters of parotids into a single structure for training.

Merging the bipolar data resulted in a compromise. A direct consequence of this compromise is a larger upper estimate boundary (e.g., see the long tail of the upper estimate in Fig. 8). This effect is because the final DVH estimate is obtained by summing the average DVH (which is calculated from both contralateral parotids and parotids that receive larger doses) with the DVHs reconstructed from the principal components.

**Figure 9:**
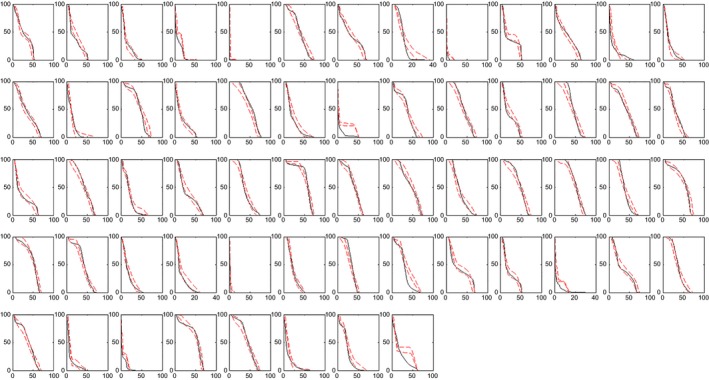
Clinical (black solid lines) and boundaries of the estimated (red dashed lines) DVHs for the mandible in 60 validation plans. The *x*‐axis of all graphs correspond to dose (Gy) while the *y*‐axis to the volume of the structure (in %).

Table 4 below summarizes the changes that were done to the first HN model along with a description of why they were made. These changes were done in multiple iterations. Table 4 shows that a total of 20 structures were removed from the model because they were causing over‐fitting. The majority of these plans resulted in over‐fitting as a result of limitations in the algorithm, such as having doses on the order of the threshold dose used to define DVHs that require in‐field regression vs those DVHs that do not (and that can be described by other DVH components, e.g., leaf transmission). Over‐fitting was particularly noticeable for the mandible, where the stepwise regression also returned terms corresponding to the product of PCS (belonging to different and orthogonal principal components). These products of PCS have no physical meaning and lead us to remove more plans in order to avoid them. A total of eight plans were excluded for the mandible and, even after these eight structures were removed from the model, over‐fitting was still taking place. This was accepted since this continuous appearance of over‐fitting suggested that multiple degrees of freedom were necessary to describe the variation in shape for the mandible DVHs. The effect of this over‐fitting can be observed on the estimated DVHs shown on Fig. 9. Figure 9 also shows the variation in DVH shape for this OAR. As can be seen from the figure, this mostly affects cases where the mandible is receiving low doses and would require planners to attempt to reduce dose to these OAR for low doses. Table 5 shows the values of statistical parameters for the HN model.

**Table 4:** Summary of changes done to the original HN model to obtain the final HN model

OAR	Number of structures available for training (remaining)	Number of geometric outliers	Number of re‐planned dosimetric outliers	Number of plans resulting in over‐fitting
Brain	94	0	0	0
Brainstem	105	0	0	0
Cord	112	0	1	3
Cord PRV	109	0	1	3
Mandible	107	0	0	8
Oral cavity	105	1	1	4
Parotids^a^	150^a^	3	2	0
Pharyngeal constrictor	77	3	3	2

a Parotids training structure includes left and right parotids, hence the large number.

**Table 5:** Values of the statistical metrics obtained for the final HN model

Structure	Regression model coefficient of determination	Regression model's parameter average chi square
Brain	0.901	1.069
Brainstem	0.883	1.049
Cord	0.681	1.033
Cord PRV	0.732	1.044
Mandible	0.896	1.075
Oral cavity	0.758	1.057
Parotid	0.829	1.05
Pharyngeal constrictor	0.683	1.039

### 3.B Validation results

**Figure 10:**
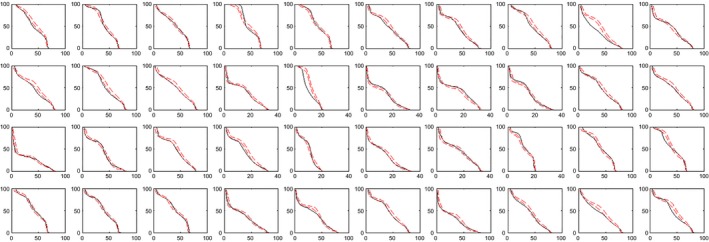
Clinical (black solid lines) and estimated (red‐dashed lines) DVHs for the rectum of 40 validation patients. The *x*‐axis of all graphs correspond to dose (Gy) while the *y*‐axis to the volume of the structure (in %).

Table 6 lists the average values of the V50, V85, and V99 metrics (positive values indicate that the estimated DVH is greater than the clinical DVH). The results indicate that the prostate model is able to generate bladder estimates within 1% on average. A similar accuracy can be achieved for rectum doses larger than 85% of the maximum dose. This is not the case in the intermediate rectum dose range, where the estimate over predicts the DVH by approximately 4%. This is likely driven by the shape of the DVH for this structure which typically has to be “bent down” to meet the clinical “V40 Gy” goal (Table 8 below). The femoral head estimates are less accurate (ranging from −2% to 3%) than those achieved for the bladder and rectum. This reduced accuracy is likely to be due to the planning technique: femoral heads are planned to remain below a maximum dose instead of well‐defined dose–volume constraints which can help to shape the DVH. In addition, the DVHs for the femoral heads exhibit steep dose fall‐offs, leading to large errors for small changes in dose. The values of Table 6 for the bladder and rectum can be compared to the results of Yuan et al.,^9^ who found estimated volumes to agree within 6% with the clinical DVHs in 71% of the cases and within 10% in 85% of the cases. The V50, V85, and V99 metrics for the bladder model of this study were within 6% in 97.5% of the cases and within 10% in 99% of the cases. The corresponding differences in the rectum were within 6% in 85% of the cases and within 10% in 93% of the cases. Figure 10 shows the clinical and estimated rectum DVHs for the 40 validation patients as an example.

**Table 6:** Prostate model average differences (±standard error of the mean) between estimated and clinical DVHs

Metric (%)	Bladder	Rectum	Femoral heads
V50	0.0 ± 0.6	4.0 ± 1.2	−2.4 ± 1.3
V85	−0.2 ± 0.2	0.9 ± 0.3	3.4 ± 0.7
V99	0.4 ± 0.1	0.7 ± 0.1	2.6 ± 0.4

Note that merging of the femoral heads into a single model structure (each femoral head was contoured separately) was done given that the treatment technique used at our center is symmetric. Merging the femoral heads into a single structure changed the mean values of the V50, V85, and V99 by values which were within the uncertainty (standard error of the mean).

**Figure 11:**
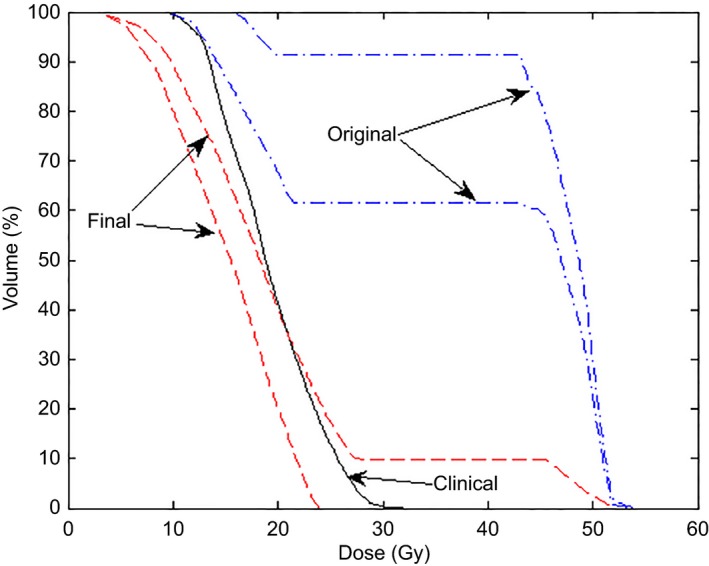
Comparison of pharyngeal constrictor DVHs estimated by the original (dash‐dotted lines) and final (dashed lines) HN model. The clinical plan (solid line) is also shown for comparison.

**Figure 12:**
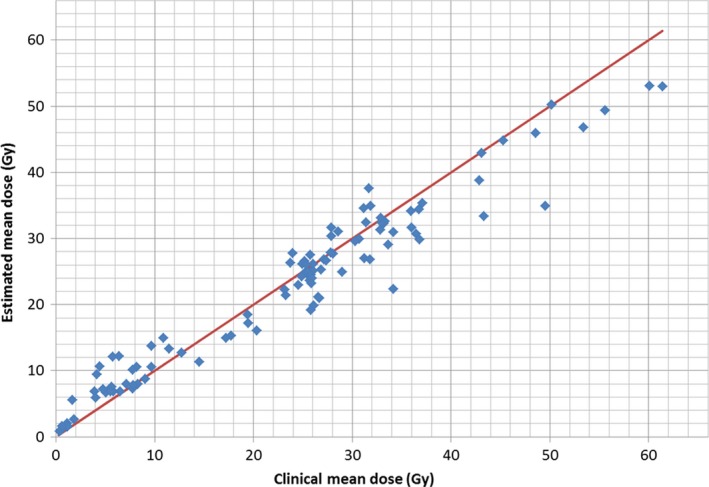
Comparison of the estimated mean doses for the parotids glands and the mean doses of the validation plans clinically used. An identity line is also shown for comparison.

Table 7 lists the average differences between the estimated and clinical DVHs for the HN model. Parotid structures were merged into a single structure for training. The results of this table show that the largest deviations happen at the V99 metric which is closely related to the maximum OAR dose. The mean difference for the other metrics ranges from −2% to 7% and, with the exception of two cases (brainstem V85 and Pharyngeal Constrictor V85) is within −2% to 4%. The reasons for the larger discrepancy close to the maximum OAR dose may be related to the long tails of the estimated DVHs (see Fig. 8 above). The pharyngeal constrictor is the least accurate structure; however, the steps taken to improve the model result increased accuracy for this OAR as shown in Fig. 11. Figure 11 shows the clinical DVH for one validation plan along with an estimated DVH from a model trained with 47 plans (including 1, 2, and 3 targets). The mean dose from the clinical plans was 19.2 Gy while that of the model created with 47 plans was 41.2 Gy. This result led us to change the creation of the HN model to include as many plans as possible as explained in Section 2.B.2. The DVH estimate obtained with the final version of the HN models is also shown in Fig. 11 and corresponds to a mean dose of 17.6 Gy. Figure 12 compares the parotids estimated and clinical mean doses which are commonly used as a clinical tolerance. The average difference between the estimated and clinical mean doses was −0.8% ± 0.4% with a standard deviation of 3.6%.

**Table 7:** HN model average differences (±standard error of the mean) between estimated and clinical DVHs

Metric (%)	Brain	Brainstem	Cord	Cord PRV	Mandible	Oral cavity	Parotid	Pharyngeal constrictor
V50	0.24 ± 0.20	0.25 ± 2.38	1.50 ± 1.48	0.02 ± 1.55	1.25 ± 1.19	5.06 ± 2.25	−2.00 ± 1.42	−0.56 ± 2.35
V85	0.08 ± 0.07	7.07 ± 2.16	0.11 ± 1.86	3.90 ± 1.39	1.23 ± 0.76	1.93 ± 1.82	3.80 ± 1.60	5.16 ± 2.90
V99	0.08 ± 0.04	6.23 ± 1.89	7.56 ± 1.31	3.73 ± 1.03	0.78 ± 0.25	2.91 ± 1.42	6.46 ± 1.17	6.61 ± 2.57

Tables 8 and 9 list the average difference between DVHs of plans generated using the models and those from the independent clinical plans. The differences were calculated for the clinical goals most commonly used (listed in the second column). The results of Table 8 show an agreement of 0.3% for the V40 Gy metric of the rectum. This is despite the reduced accuracy of the V50 metric for the estimated DVH. This is because the line objectives are placed on the lower boundary of the estimate which coincides with the clinical DVH in many cases (see Fig. 10). The V50 metric on the other hand, was calculated based on the estimated average. Table 9 only shows the results for the highest dose PTV since the results for the other PTVs (intermediate and low doses) agreed within 1.2%. Table 9 also includes results for structures which were not trained due to insufficient data (structures which are contoured less frequently) and optical structures (which were also excluded from the model). The DVH metrics for the PTV agreed within 2.0% while the agreement for the OARs included in the model range from −1.6% to 2.5%. The table also shows reduced agreement for untrained OARs.

**Table 8:** DVH differences between clinical plans and those obtained using the prostate model

Structure	Clinical goal	Difference	
		Average	Standard deviation
PTV	D99% (%)	0.32	1.01
	V105% (cm^3^)	−1.95	8.88
Bladder^a^	V80 Gy ≤ 15%	−0.04	0.75
	V75 Gy ≤ 25%	0.16	0.43
	V70 Gy ≤ 35%	0.09	0.29
	V65 Gy ≤ 50%	−1.61	6.85
	V40 Gy ≤ 70%	−0.54	3.15
Rectum^a^	V75 Gy ≤ 15%	0.31	0.94
	V70 Gy ≤ 20%	0.26	0.94
	V65 Gy ≤ 25%	−0.97	3.77
	V60 Gy ≤ 35%	0.05	1.59
	V50 Gy ≤ 50%	−0.26	3.02
	V40 Gy ≤ 55%	0.27	3.96
Femoral heads^a^	V53 Gy ≤ 5%	0.00	0.00
	V50 Gy ≤ 10%	−0.01	0.04

a Trained structure.

**Table 9:** DVH differences between clinical plans and those obtained using the HN model

Structure	Clinical goal	Difference	
		Average	Standard deviation
High‐dose PTV	D99% > 93% of Rx	−0.05	3.55
	D95% > 100% of Rx	0.24	2.01
	D15% < 107% of Rx	1.83	3.07
	Dmean < 103% of Rx	1.41	2.68
Brain^a^	Dmax < 70 Gy	1.41	6.49
Brainstem^a^	Dmax < 54 Gy	0.56	4.19
Cervical esophagus	Dmean < 40 Gy	4.12	9.72
Cord^a^	Dmax < 40 Gy	−0.18	4.25
Cord PRV^a^	V45 Gy < 1%	0.69	2.33
Glottic larynx	Dmean < 35 Gy	2.64	6.72
Lens	Dmax < 10 Gy	0.38	3.2
Mandible^a^	Dmax < 70 Gy	1.97	4.83
Optic chiasm	Dmax < 45 Gy	0.4	3.77
Optic nerve	Dmax < 45 Gy	−0.47	3.15
Oral cavity^a^	Dmean < 40 Gy	2.52	11.32
Orbit	Dmax < 45 Gy	1.01	3.32
Parotid^a^	Dmean < 26 Gy	−1.58	4.65
Pharyngeal constrictor^+^	Dmean < 45 Gy	0.98	4.12

a Trained structure.

## 4 DISCUSSION

This paper summarized the process used to build models with emphasis on the approach used to address outliers. In terms of KBP model creation, there is currently no consensus on proper outlier identification and mitigation. Delaney et al. investigated the effect of dosimetric outliers in the creation of KBP models and their results suggested that dosimetric outliers have minimal effect on the accuracy of KBP models, instead they affected their precision.^19^ Given that the models described are available to a potentially large number of users, the creation of robust models was desirable by removing influential outliers while keeping plans that provide additional information. This included the desire to create models which are both accurate and precise, hence the need to re‐plan dosimetric outliers. The models presented in this manuscript were created following a process which required multiple iterations that have to be done manually by the user and is, hence, time consuming. This made it impractical to evaluate the effect that an individual outlier would have on the models. Our results, however, show that the steps taken to create the models improved the accuracy of DVH estimates and Fig. 11 shows an example of it. The time and effort required to address outliers is likely dependent on each institution's planning practice; as institutions with highly standardized planning techniques are likely to have less dosimetric outliers.

The analysis on this paper focused on the accuracy of the estimated DVHs and our results show to be comparable to those of previous tools.^9^ The models in this study were compared by calculating the difference between the volume of the DVH curve in the clinical plan and the volume of the estimated DVH. These volumes were compared at three points along the x axis which corresponded to the 50%, 85% and, 99% of the maximum dose (of the clinical plan). These points were used in a previous publication^9^ and provided a benchmark for model comparison. The selected metrics can also be generalized to plans with different prescriptions, where many clinical goals would be irrelevant. Another advantage of the metrics used in this study can be seen in the planning results for the femoral heads (Table 8). Table 8 shows nearly perfect agreement but is due to fact that the femoral heads maximum dose constraints were easily met and the accuracy of the KBP model would not be accurately quantified. Note that the V50 metric used in this study also provides a point to compare the dose at an intermediate point along each DVH. This is useful to evaluate what happens with OARs whose constraints are specified in terms of maximum dose, where DVHs with different shapes (which would be an incorrect estimate) could yield the same clinical metric (e.g., maximum dose). This could be missed if clinical objectives are the only metrics used to evaluate models. It should be noted that other metrics have been used by other investigators. Appenzoller et al., for example, used the sum of residuals^8^ while Tol et al. used a combination of clinical objectives to evaluate the performance of their models.^18^ There is, however, no evidence that one metric is better than the other and this topic needs further investigation. The modified V50, V85, and V99 metrics used in this study can be used to evaluate the accuracy of estimated DVHs for all plans irrespective of dose prescription and provide a reasonable way to quantify the shape of the estimated DVH.

The models were also used as optimization objectives as part of the validation process. It should be noted that while some authors have benchmarked their models by using them to guide the optimization process,^18^ others have focused entirely on the accuracy of the estimated DVHs,^9^, ^21^ and others in both.^8^ While the need to re‐plan all plans remains debatable, we decided to use our models as optimization objectives for the validation process. This provided an end‐to‐end test for the models and ensures that the model does not compromise on untrained structures. The models were therefore evaluated in three different ways: (a) by analyzing the accuracy of the estimated DVHs on the training set (summarized in the goodness of fit statistics), (b) by analyzing the accuracy of estimated DVHs on an independent validation set, and (c) by using the estimated DVH to guide the inverse planning process. This therefore resulted in a rigorous model commissioning approach that gives confidence of its performance.

Planning can be done in a variety of ways, potentially leading to different results. Therefore, the planning of this study was done by means of an automated run without user interaction. This approach leads to user independent results and is more practical (due to the large number of validation patients). However, the approach is limited since the presence of untrained OARs could not be accounted for. Our results show that the models can be used by users outside of the institution where they were originally created yet preserve the planning trend. Additional planning could be conducted for cases where differences between the estimated and the original DVHs are significant since it implies potential for dose reduction (cases where the DVH was under‐estimated) or increased dose (cases the DVH was over‐estimated). The question of what difference is considered significant to require additional planning is still unknown and is beyond the scope of this publication.

The presence of bipolar data was evident in the bladder of the prostate model and in the parotids of the HN model. The simplest way to address bipolar data is to simply separate them as was done in the prostate model. The bipolar data for the parotids is due to ipsi‐ and contra‐lateral parotids and its separation is more challenging as this would require a user to come up with a strategy to accurately identify these groups of parotids. Incorporation of techniques that aid in cluster classification could improve the accuracy of parotid DVH estimates.^22^

## 5 CONCLUSIONS

The creation of KBP models requires diligence since the presence of outliers can affect the accuracy of the estimated DVHs. A combination of multiple tools should be used to identify and address outliers. The process followed to create models presented in this publication led to DVH estimates with an accuracy of −2%–4% for the prostate model and from −2% to 8% for the HN model with agreements in parotid mean doses off less than 1% on average. More automated ways to analyze model creation would be desirable and would allow investigators to handle outliers that would have a negative effect on a model.

## CONFLICT OF INTEREST

This work was funded by Varian Medical Systems.

## NOTES

*Varian Medical Systems, “Eclipse Photon and Electron Algorithms Reference Guide”, 2014.

^†^Varian Medical Systems, “CancerCare Manitoba Head and Neck Model Description”, P1009012‐002‐B, 2014.

## References

[1] Yuan L , Wu QJ , Yin F , et al. Standardized beam bouquets for lung IMRT planning. Phys Med Biol. 2015;60:1831–1843. 25658486 10.1088/0031-9155/60/5/1831 PMC4384508

[2] Lee T , Hammad M , Chan TCY , Craig T , Sharpe MB . Predicting objective function weights from patient anatomy in prostate IMRT treatment planning. Med Phys. 2013;40:121706. 24320492 10.1118/1.4828841

[3] Liu J , Wu QJ , Kirkpatrick JP , Yin F‐F , Yuan L , Ge Y . From active shape model to active optical flow model: a shape‐based approach to predicting voxel‐level dose distributions in spine SBRT. Phys Med Biol. 2015;60:N83–N92. 25675394 10.1088/0031-9155/60/5/N83

[4] Shiraishi S , Moore KL . Knowledge‐based prediction of three‐dimensional dose distributions for external beam radiotherapy. Med Phys. 2016;43:378–387. 26745931 10.1118/1.4938583

[5] Shiraishi S , Tan J , Olsen LA , Moore KL . Knowledge‐based prediction of plan quality metrics in intracranial stereotactic radiosurgery. Med Phys. 2015;42:908–917. 25652503 10.1118/1.4906183

[6] Wu B , Ricchetti F , Sanguineti G , et al. Patient geometry‐driven information retrieval for IMRT treatment plan quality control. Med Phys. 2009;36:5497–5505. 20095262 10.1118/1.3253464

[7] Zhu X , Ge Y , Li T , Thongphiew D , Yin F‐F , Wu QJ . A planning quality evaluation tool for prostate adaptive IMRT based on machine learning. Med Phys. 2011;38:719–726. 21452709 10.1118/1.3539749

[8] Appenzoller LM , Michalski JM , Thorstad WL , Mutic S , Moore KL . Predicting dose‐volume histograms for organs‐at‐risk in IMRT planning. Med Phys. 2012;39:7446–7461. 23231294 10.1118/1.4761864

[9] Yuan L , Ge Y , Lee WR , Yin FF , Kirkpatrick JP , Wu QJ . Quantitative analysis of the factors which affect the interpatient organ‐at‐risk dose sparing variation in IMRT plans. Med Phys. 2012;39:6868–6878. 23127079 10.1118/1.4757927

[10] Das IJ , Cheng C‐W , Chopra KL , Mitra RK , Srivastava SP , Glatstein E . Intensity‐modulated radiation therapy dose prescription, recording, and delivery: patterns of variability among institutions and treatment planning systems. J Natl Cancer Inst. 2008;100:300–307. 18314476 10.1093/jnci/djn020

[11] Nelms BE , Robinson G , Markham J , et al. Variation in external beam treatment plan quality: an inter‐institutional study of planners and planning systems. Pract Radiat Oncol. 2012;2:296–305. 24674168 10.1016/j.prro.2011.11.012

[12] Wu B , McNutt T , Zahurak M , et al. Fully automated simultaneous integrated boosted‐intensity modulated radiation therapy treatment planning is feasible for head‐and‐neck cancer: a prospective clinical study. Int J Radiat Oncol Biol Phys. 2012;84:e647–e653. 22867890 10.1016/j.ijrobp.2012.06.047

[13] Good D , Lo J , Lee WR , Wu QJ , Yin FF , Das SK . A knowledge‐based approach to improving and homogenizing intensity modulated radiation therapy planning quality among treatment centers: an example application to prostate cancer planning. Int J Radiat Oncol Biol Phys. 2013;87:176–181. 23623460 10.1016/j.ijrobp.2013.03.015

[14] Lian J , Yuan L , Ge Y , et al. Modeling the dosimetry of organ‐at‐risk in head and neck IMRT planning: an intertechnique and interinstitutional study. Med Phys. 2013;40:121704. 24320490 10.1118/1.4828788 PMC3838428

[15] Moore KL , Brame RS , Low DA , Mutic S . Experience‐based quality control of clinical intensity‐modulated radiotherapy planning. Int J Radiat Oncol Biol Phys. 2011;81:545–551. 21277097 10.1016/j.ijrobp.2010.11.030

[16] Fogliata A , Belosi F , Clivio A , et al. On the pre‐clinical validation of a commercial model‐based optimisation engine: application to volumetric modulated arc therapy for patients with lung or prostate cancer. Radiother Oncol. 2014;113:385–391. 25465726 10.1016/j.radonc.2014.11.009

[17] Fogliata A , Wang P , Belosi F , et al. Assessment of a model based optimization engine for volumetric modulated arc therapy for patients with advanced hepatocellular cancer. Radiat Oncol. 2014;9:236. 25348465 10.1186/s13014-014-0236-0 PMC4219039

[18] Tol JP , Delaney AR , Dahele M , Slotman BJ , Verbakel WFAR . Evaluation of a knowledge‐based planning solution for head and neck cancer. Int J Radiat Oncol Biol Phys. 2015;91:612–620. 25680603 10.1016/j.ijrobp.2014.11.014

[19] Delaney AR , Tol JP , Dahele M , Cuijpers J , Slotman BJ , Verbakel WFAR . Effect of dosimetric outliers on the performance of a commercial knowledge‐based planning solution. Int J Radiat Oncol Biol Phys. 2016;94:469–477. 26867876 10.1016/j.ijrobp.2015.11.011

[20] Rawlings JO . Applied Regression Analysis: A Research Tool. Belmont, California: Wadsworth, Inc.; 1988.

[21] Boutilier JJ , Craig T , Sharpe MB , Chan TCY . Sample size requirements for knowledge‐based treatment planning. Med Phys. 2016;43:1212–1221. 26936706 10.1118/1.4941363

[22] Yuan L , Wu QJ , Yin F‐F , Jiang Y , Yoo D , Ge Y . Incorporating single‐side sparing in models for predicting parotid dose sparing in head and neck IMRT. Med Phys. 2014;41:021728. 24506619 10.1118/1.4862075 PMC3977781

